# Failure to detect Schmallenberg virus RNA in ram semen in the UK (2016–2018)

**DOI:** 10.1002/vro2.39

**Published:** 2022-06-20

**Authors:** Alice Curwen, Scott Jones, Ceri Stayley, Laura Eden, Heather McKay, Peers Davies, Fiona Lovatt, Stephen Dunham, Rachael Tarlinton

**Affiliations:** ^1^ School of Veterinary Medicine and Science University of Nottingham Leicestershire UK; ^2^ Department of Life Sciences Imperial College London London UK; ^3^ Bishopton Veterinary Group, Ripon North Yorkshire UK; ^4^ Three Valleys Veterinary Irvinestown Enniskillen UK; ^5^ Department of Livestock & One Health University of Liverpool Liverpool UK

## Abstract

**Background:**

Schmallenberg virus (SBV) is a midge‐borne arbovirus that first emerged in the European ruminant population in 2011 and has since settled to an endemic pattern of disease outbreaks on an approximately 4‐year cycle when herd immunity from the previous circulation drops to a point allowing renewed widescale virus circulation. The impacts of trade restrictions on genetic products (semen, embryos) from affected areas were severe, particularly after the discovery that the virus is intermittently shed in the semen of a small number of bulls. The trade in small ruminant (ram and goat) semen is less than that of bulls; nonetheless, there has been no study into the shedding rate of SBV in ram semen.

**Methods:**

Semen samples (*n* = 65) were collected as part of UK ram trials and artificial insemination studies around the period of the 2016–2018 SBV recirculation. Semen was preserved in RNA*later* for shipping, and RNA extraction with RNeasy and S gene RT‐quantitative PCR performed for SBV nucleic acid detection.

**Results:**

No SBV RNA was detected in any samples.

**Conclusions:**

While larger numbers of animals would be needed to completely exclude the possibility of SBV shedding in ram semen, this trial nonetheless highlights that this is likely a rare event if it occurs at all and is unlikely to play a role in disease transmission.

## INTRODUCTION

Schmallenberg virus (SBV) is a novel *Orthobunyavirus* that was first identified in Germany in late 2011 via metagenomic analysis of bovine blood.^1^ Three months after the initial outbreak, England reported cases of SBV during 2012–2013.^2^ A re‐emergence of SBV occurred in the UK, Ireland and Belgium during 2016–2018^3^, ^4^, ^5^; then more recently in Germany, Denmark and England in 2020–2021.^6^, ^7^, ^8^ Insect vectors belonging to certain species of *Culicoides* biting midge are responsible for SBV transmission.^9^ Domestic ruminants are the main hosts for the virus in Europe, specifically cattle, sheep and goats; however, the presence of SBV in deer, camelids and a variety of wild ruminants has been reported.^2^

Infection in adult cattle generally causes mild signs, including reduced milk yield, fever and diarrhoea, whereas adult sheep appear subclinical/asymptomatic.^10^, ^11^, ^12^ The greatest clinical impact of SBV is observed when naïve pregnant animals become infected during a susceptible period of gestation (approximately days 16–173 in cattle and days 28–56 in sheep).^13^ Transplacental infection ensues, resulting in abortions, stillbirths and congenital malformations termed arthrogryposis‐hydranencephaly syndrome.^14^ Infection during early gestation probably downregulates interferon tau production (the maternal recognition of pregnancy signal in ruminants), causing premature embryo loss and return to oestrus.^15^, ^16^, ^17^ To date, large outbreaks of clinical disease have been observed on a roughly 4‐year cycle in Europe, consistent with modelling estimating that this is roughly the time natural seropositivity from the previous outbreak would be negligible due to turn over of animals in normal ovine production systems in Europe.^18^

The European livestock sector incurred severe economic losses due to SBV. Major trade restrictions were implemented after the SBV outbreak, with exports of live animals and genetic products (breeding stock, embryos and semen) from affected countries being blocked by many countries.^19^ In this context, potentially infectious SBV RNA found in bull semen has been reported by multiple groups to be intermittently shed for up to 3 months postinfection.^20^, ^21^, ^22^ To be declared SBV‐free, semen samples collected after June 2011 should be analysed via an approved RNA extraction and RT‐quantitative PCR (RT‐qPCR) method, unless the animal tested negative for SBV‐specific antibodies a minimum of 28 days post‐semen production.^23^ While the presence of SBV RNA in semen does not automatically indicate venereal transmission^24^ or any effect on the embryo or successful conception, this is thought to be the mechanism by which the re‐emergence of bluetongue virus (BTV) in Europe occurred (BTV is a ruminant arbovirus that can cause cross‐placental infections) via the use of frozen reproductive materials.^25^, ^26^

A focus has been placed on investigating SBV in bull semen, particularly as 50% of the world's bulls enrolled in semen collection programmes are located in Europe.^27^ Multiple studies have reported that SBV RNA is intermittently shed in bull semen.^20^, ^21^, ^22^, ^28^ One experimental study reported no detection of SBV RNA in semen from two bucks (male goats) infected with SBV;^29^ similarly, one experimental study in sheep detected no SBV RNA in the testicles of the two entire rams. However, to the best of the authors’ knowledge, no exploration into SBV shedding in ram semen has been conducted. As the sheep breeding season is so compact compared to all‐year‐round dairy calving, the impact of SBV outbreaks during the breeding season on European ovine flocks may be high. French farmers observed a higher median frequency of morbidity in lambs (8%) versus 3% for calves and 2% for caprine kids.^17^ Therefore, this study aimed to investigate the potential shedding of SBV RNA in ram semen samples collected from around Great Britain. Semen was obtained during and after the 2016–2018 outbreak from rams participating in the ‘RamCompare’ breeding trials or artificial insemination studies. Testing for SBV viral RNA was conducted using established qPCR methods.^30^

## MATERIALS AND METHODS

The re‐use of samples collected during the ‘RamCompare’ trial and semen from animals participating in a study of sexual transmission of Maedi Visna was given ethical approval by the University of Nottingham School of Veterinary Medicine and Sciences committee for animal research and ethics. The original Maedi Visna trial was conducted under UK Home Office, Animals (Scientific Procedures) Act 1986 licence no. PPL 30/3367.

Semen was collected between October 2016 and August 2018; seven samples were collected during September 2016, 24 samples were collected between July and August 2017, and 34 samples were collected from July to September 2018. These samples were from rams of 12 breeds (Abermax [5], Aberfield [3], Texel [11], Suffolk [11], Hampshire Down [11], Charollais [12], Meatlinc [6], Blue Texel [1], Southdown [2], Dorset Down [1], Blue du Maine [1], Beltex [1]) ranging from >6 months (*n* = 15), 1 year old (*n* = 9), 2 years old (*n* = 15), 3 years old (*n* = 15) to 4+ years old (*n* = 7) across Great Britain, producing 65 samples (Table 1). The majority of the rams were enrolled in the ‘RamCompare’ estimated breeding value trials and we did not have access to their flock history with respect to SBV. Two rams (both Aberfield) sampled in 2016 were participating in an artificial insemination trial in a flock that seroconverted during summer 2016, the results of which have been reported.^31^

**TABLE 1:** Details of ram semen samples analysed

Year/season of collection	Number of samples
October 2016	7
July 2017	11
August 2017	13
July 2018	18
August 2018	11
September 2018	5
Total	65

**FIGURE 1:**
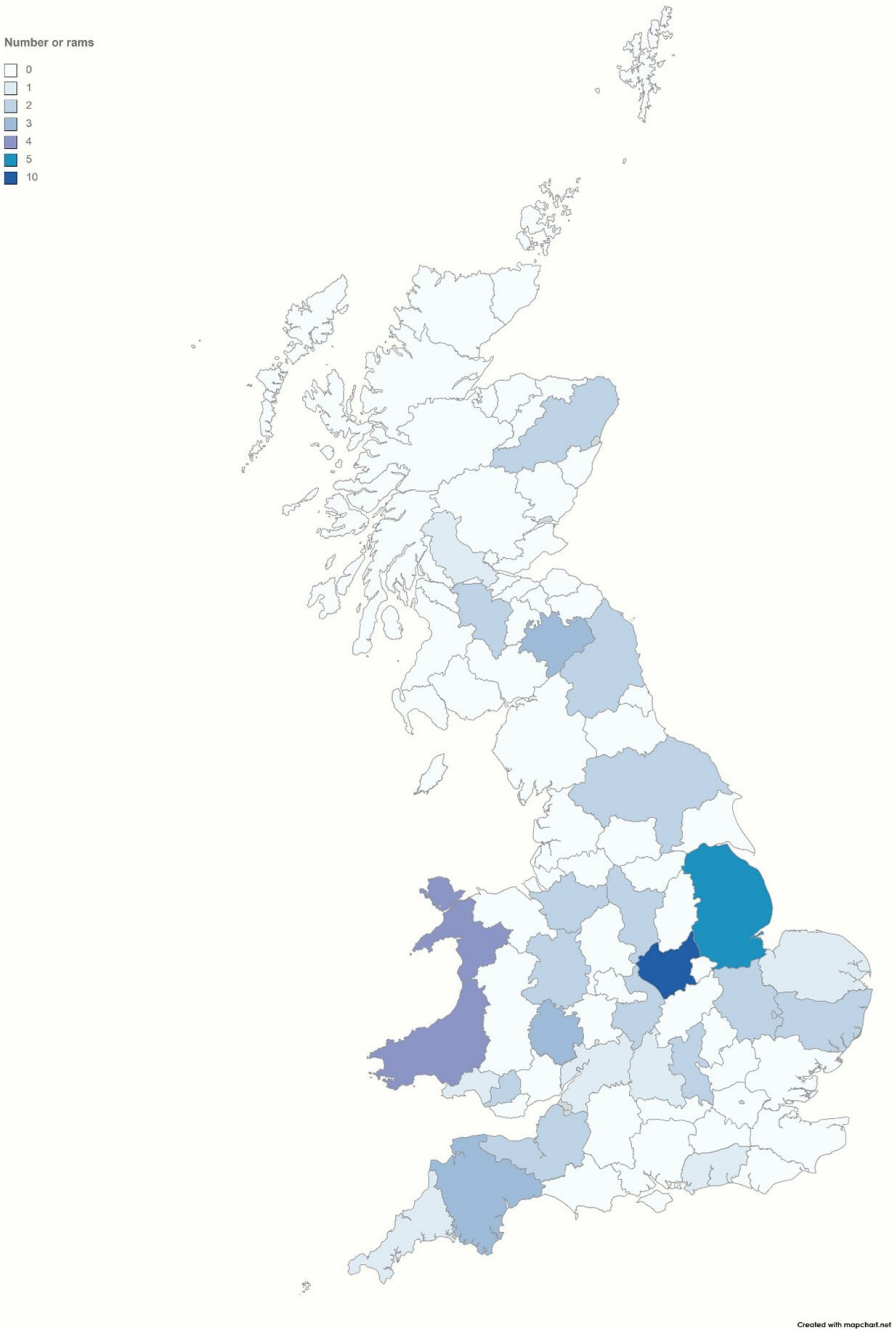
Spatial distribution of ram semen sample locations by county in Great Britain during the sampling period 2016–2018 (created using mapchart; https://www.mapchart.net/uk.html). Counties are coloured according to the number of rams sampled, with colour intensity increasing with a greater number of rams sampled

To ensure anonymity for the farmers who contributed to the study, the geographical location was set to the county level (Figure 1). Manually collected fresh semen was preserved in RNA*later* (3× volume of RNA*later* [Sigma—Aldrich] to one volume of semen), transported at room temperature to the University of Nottingham and then stored at −20°C until analysis. According to the manufacturer's instructions, RNA was extracted from the semen samples using the RNeasy Mini Kit (Qiagen). cDNA synthesis was performed with Moloney murine leukaemia reverse transcriptase(Promega) following the manufacturer's protocol.

qPCR was performed using a published protocol (Table 2).^30^ The LightCycler 480 system (Roche) was used to perform reactions, which were run in duplicate. The reaction conditions consisted of 12.5 μl of LightCycler 480 probe master mix (Roche), 4.5 μl qPCR‐grade water, 1 μl SBV FAM probe at 0.075 pmol/μl, 1 μl of each primer at 0.4 pmol/μl and 5 μl template cDNA, giving a total reaction volume of 25 μl. The cycling conditions for this reaction consisted of an initial denaturation at 95°C for 15 min, followed by 45 cycles of 95°C for 15 s, 55°C for 30 s and 72°C for 30 s.

**TABLE 2:** Primers and probes used in this study

Primer target	Primer sequence^a^	Product length (bp)	Accession number^b^
Schmallenberg virus	F (5′‐TCAGATTGTCATGCCCCTTGC −3′)	88	KC355459
	R (5′‐TTCGGCCCCAGGTGCAAATC −3′)		
	P [6FAM]TTAAGGGATGCACCTGGGCCGATGGT[TAM]		
Ovine β actin	F (5′‐ GTCACCAACTGGGACGACA‐3′)	208	U39357
	R (5′‐AGGCGTACAGGGACAGCA −3′)		

^a^ F stands for forward (sense), R for reverse (anti‐sense) and P for the Schmallenberg virus probe.

^b^ GenBank accession numbers.

PCR was used to amplify the SBV small (S) segment from an SBV cell culture isolate (kindly provided by the Friedrich Loeffler Institute, Greifswald, Insel Reims, Germany), which was then used to prepare a serial dilution for inclusion in each assay. Standard curves for quantitative analysis were run on each plate and analysed using LightCycler 480 software. To confirm that sample quality was adequate for qPCR, β actin end point PCR was performed on all samples and only samples that amplified β actin were included in the analysis. Primers for this quality control step were taken from a previously described assay with reaction conditions as follows: 10 μl One*Taq* 2× master mix with standard buffer (New England BioLab), 7.2 μl qPCR‐grade water, 0.4 μl of each primer at a 0.5 pmol/μl and 2 μl template cDNA giving a final reaction volume of 20 μl.^24^ The cycling conditions consisted of 95°C for 30 s followed by 45 cycles of 95°C for 20 s, 58°C for 20 s and 72°C for 30 s; the primer sequences are shown in Table 2.

To confirm that the extraction method used was adequate for the detection of SBV RNA in ram semen with RNA*later* preservative. A pooled semen sample (three rams) was spiked with cultured SBV (2.67 × 10^6^ TCID_50_/ml) as previously described;^31^ then, RNA extraction in triplicate and qPCR (in triplicate on each extraction) were performed as described above. To compare extraction methods, pooled spiked semen in triplicate was also extracted with TRIzol LS (Invitrogen) and either isopropanol precipitation as per the manufacturer's instructions or magnetic bead precipitation (Agencourt AMPure XP kit, Beckman Coulter) as per the manufacturer's instructions.

## RESULTS

A total of 65 individual animal samples from 12 different ram breeds across Great Britain were used to screen for SBV in ram semen. Of the 65 samples collected between 2016 and 2018, no SBV RNA was detected in any ram semen. To validate sample quality for qPCR, only samples that amplified ovine β actin from cDNA were included in this study.

The spiking experiment with cell culture SBV demonstrated an average Ct value in the final qPCR of 25.97 for the Qiagen kit extraction; of 30.69 for the TRIzol LS reagent and isopropanol method; with failure to detect SBV at all with the Agencourt magnetic bead kit; demonstrating that the assay was able to detect SBV reliably in this substrate and that the Qiagen extraction kit detected virus at a higher sensitivity (lower Ct value reflective of higher viral copy number) than the TRIzol LS method.

## DISCUSSION

Despite collection occurring during and after the 2016/2017 disease outbreak over a wide geographical area of Great Britain,^31^ no SBV RNA was detected in ram semen at any stage. This does not completely eliminate the possibility that a small number of rams may shed viral RNA in semen.

Two of the seven rams sampled during October 2016 (both Aberfield) had semen samples collected at a time point of 2 days apart; both animals yielded negative SBV RNA results each time. These rams were also part of an SBV viral neutralising antibody study that found blood samples taken from 13 rams (seven semen samples in this study) displayed increases in antibody titres during October 2016. This suggested potential recent exposure to the virus during the 2016 breeding season,^31^ yet no SBV RNA was detected in the semen of those rams. SBV has a relatively short viraemic period (2–6 days)^19^; however, bulls that displayed seroconversion (2–4 weeks postinfection) also had consecutive positive SBV RNA in their seminal cell fraction,^20^ eluding the testis as a potential privilege site for SBV infection and transmission. Nonetheless, it is yet to be tested whether excreted SBV RNA is capable of transmitting and causing infection. Monitoring for seroconversion via ELISA could indicate which males are at risk for semen excretion of SBV, although as it appears to be low numbers of animals this would likely be more applicable to semen export situations rather than natural mating in SBV endemic areas.

The results obtained in this study indicate that the risk of transmitting SBV RNA venereally via rams is low, suggesting that a major role in natural disease transmission or persistence in Europe is unlikely to occur due to ram semen. However, it is important to answer these questions, as other vector‐borne viruses have been detected in human semen long after infection. Infectious Zika virus was found in human semen up to 69 days postexposure,^32^ with viral RNA detected up to 414 days post‐sign onset.^33^ Ebola virus RNA was also discovered in human semen 6 months postinfection, with a maximum duration of viral RNA still seen at 696 days; however, it is not known if this would lead to infectious venerial transmission.^34^

It is possible that our assay failed to detect the variants circulating in 2016–2018 due to sequence variation in circulating SBV virus. However, there does not appear to have been significant variation in the S gene sequence (which the qPCR assay used here targets) that would have resulted in assay failure at this time; indeed, the virus seems remarkably stable and we used the same assay to detect SBV viraemia in sheep in 2016.^6^, ^31^ Alternative assays have been proposed to detect the virus (particularly in the M segment),^35^ which is where the major hypervariable region in SBV is reported. Such variation is, however, likely due to within animal tissue specificity/tropism and mutation; it is not reflected in circulating viruses where selection pressure to maintain transmissibility in multiple mammalian and insect hosts is high.^36^ The performance for the S segment in the assay compared with the M gene assays was similar.^35^ Consequently, it seems unlikely that qPCR assay mismatches were responsible for the failure to detect SBV in ram semen.

Our findings are in contrast to those in bulls where 11/95 bulls (29 semen batches),^20^ three bulls out of seven (29/136 semen batches) and 5/100 bulls^22^ and 7/131 bulls^37^ temporarily excrete SBV RNA in their semen. This may be due to a lack of sampling power because our study sample size was small. Using a power calculation^38^ of the number of rams required to reliably detect the rate of SBV shedding in semen observed in cattle^20^ would require 379 rams. In a bovine study,^20^ a small number of bulls tested positive (11/95), with six of the 11 bulls testing positive on multiple occasions, shedding SBV RNA in their semen from 3 days to 8 weeks postinfection. The finding of intermittent shedding in a small number of bulls has been reported in other studies.^22^ Our samples were mainly single time point collections and not continuous monitoring of individuals, so this could have missed intermittent shedding.

Previous studies have recommended RNA extraction for the detection of SBV RNA from semen samples in bulls using TRIzol LS reagent and the Magmax (Applied Biosystems magnetic beads) precipitation method.^20^, ^22^, ^39^ This method can only be performed using a Kingfisher (Thermoscientific) robotic extraction system and this method was not available to the authors. We could not exactly replicate this method, and the magnetic bead precipitation method we used, with the recommended TRIzol LS reagent, failed completely. When we substituted isopropanol precipitation, the TRIzol LS reagent still underperformed in terms of viral RNA extraction efficiency when compared to the Qiagen kit reported herein. Interestingly, one study,^37^ using nonautomated systems for bull semen, reported, similar to our study, greater sensitivity with the Qiagen kit. That study postulated that the different sample preparations (including different semen extenders and dilutions) may affect the extraction efficiency of specific kits. It is important to consider that the template used here (ram semen in RNA*later*) was not the same protein composition as commercial straws of bull semen used in the EU reference laboratory ring trial.^39^ Consequently, there is no guarantee that the extraction efficiency of different reagents will not display species‐ and substrate‐specific differences.

Our results are consistent with two experimental studies using entire rams and bucks,^12^, ^29^ while one of these studies only had two entire males tested,^29^ and the other seven animals,^12^ SBV RNA was not detected in testicles or semen in either study. In the absence of any other data on semen shedding in rams, we present these results demonstrating that this is, at least, not a common occurrence even during periods of active virus circulation in ruminants.

## CONFLICTS OF INTEREST

The authors declare they have no conflicts of interest.

## ETHICS STATEMENT

The authors confirm that the ethical policies of the journal, as noted on the journal's author guidelines page, have been adhered to. The research was approved by the University of Nottingham School of Veterinary Medicine and Sciences committee for animal research and ethics.

## AUTHOR CONTRIBUTIONS

AC, SJ, CS, HM, LE performed laboratory work. PD, FL collected and coordinated samples. AC wrote the manuscript and RT and SD reviewed the manuscript and supervised SJ. RT managed and corordinated the project.

## Data Availability

The data that support the findings of this study are available within the article.
